# Hearing Loss in Older Adults: Implications for Cognitive Load and Brain Structure and Function

**DOI:** 10.1093/geroni/igaa057.2882

**Published:** 2020-12-16

**Authors:** Frank Lin

**Affiliations:** Johns Hopkins University School of Medicine, Baltimore, Maryland, United States

## Abstract

Age-related hearing loss is prevalent in two-thirds of older adults and reflects progressive impairments in cochlear function leading to impoverished peripheral neural encoding of sound. Research has demonstrated the broader implications of hearing loss for the health and functioning of older adults, particularly with respect to brain aging and dementia. This presentation will summarize current epidemiological and neuroimaging evidence for how hearing loss in older adults affects cognitive load and brain structure/function and relate this contemporary research with previous psychological studies proposing ‘information degradation’ and ‘sensory deprivation’ hypotheses of how hearing may affect cognitive function. Finally, the design of an ongoing NIA-funded randomized controlled trial (ACHIEVE- Aging and Cognitive Health Evaluation in Elders) that will determine if hearing treatment reduces the risk of cognitive decline, dementia, and brain aging in adults will be discussed. Part of a symposium sponsored by Brain Interest Group.

